# Chronic Nasal Diphtheria

**Published:** 1910-07-02

**Authors:** Philip A. Harry


### Resident Medical Officers' Department.

### CHRONIC NASAL DIPHTHERIA.

By PHILIP A. HARRY, M.D., D.P.H.

This important condition is apt to remain undiagnosed until attention is directed to it, notwithstanding the fact that it is easily recognisable from other types of rhinitis, and bacteriologically a growth on blood serum soon confirms the diagnosis. The affection should perhaps be called simple nasal diphtheria, seeing that the majority of cases commence insidiously without any acute symptoms, and do not follow directly on any other form of diphtheria.

The characteristic feature is a thin, unirritating serous discharge from one nostril or both. The discharge is at times blood-stained; in this it differs from the mucoid secretion of acute rhinitis, which is more opaque and gelatinous. A thin, watery discharge is also associated with the chronic rhinitis, occasionally a symptom of late hereditary syphilis, but in this case there is usually excoriation of the skin around the nostril. This raw appearance is not noticed in simple nasal diphtheria, nor is there the formation of pustules and scabs on the upper lip associated with eczematous blepharitis and rhinitis.

Of the thirty-five cases treated during the last twelve months all were children under ten; the majority were between three and seven.

They are usually brought up with a history of persistent nasal discharge and earache. The mothers very rarely pay any attention to nasal discharge, even if profuse; it is only when earache becomes an associated symptom that the condition is considered important. The mother may add that the child has become dull and listless. There is no history of fever or any other acute symptom. Anorexia is not marked. The children usually attend school up to the time of seeking advice.

In some of those who were treated as in-patients there was a tendency for the temperature to rise slightly in the evening, but only if they were allowed up and had been excessively energetic during the day. There is never the appearance of acute coryza, although the mother will reply in answer to our anxious inquiries that she thought it was only a "cold in the nose."

The earache comes on insidiously; it does not appear to bear any relation to the quantity or duration of the nasal discharge. The pain is a dull aching, worse at night; it may be severe enough to produce any of the manifestations that accompany an acute otitis media. With regard to the nasal discharge, it is even more difficult to get a definite statement as to its commencement.

Sooner or later an ear discharge is noticed; it bears a close resemblance to that from the nose—it is thin, serous, and often blood-stained, and gives a pure growth of the diphtheria bacillus. On inspection the turbinates, especially the inferior, are swollen, but not enough to obstruct the passage of air. The characteristic secretion covers the whole mucous membrane, and gives it a moist, glistening appearance, not the appearance of an acute inflammatory condition. Even gentle manipulations, such as "taking a swab" of the discharge, will produce a slight blood-staining of the fluid. A membrane is never noticed in these chronic cases.

Portions of the mucous membrane removed from the turbinates and suitably stained nearly always show the organisms in close relationship to the mucous glands, at some distance beneath the columnar epithelium. There is marked engorgement of the vessels in the submucosa.

In many instances the infection can be traced to a case of acute membranous diphtheria; in others neither the patient himself nor any members of the family have had diphtheria. In one case a younger brother died of membranous croup six months before the patient was brought with nasal discharge and earache. Another child was treated at a fever hospital for scarlet fever, and while there developed diphtheria. Three months after her discharge she was brought up with the characteristic nasal discharge, which in this case was noticeably unilateral. Examination showed a boot button in the inferior meatus. The child then confessed that she had put the foreign body into her nose while she was in the fever hospital. The appearance of the button certainly confirmed her statement. In a few instances these patients have proved sources of serious infection to others. The younger sister, aged nine months, of a child suffering from chronic nasal diphtheria was admitted with purulent ophthalmia; as soon as the organism was found to be the diphtheria bacillus she was removed to the fever hospital. Death took place a week afterwards from laryngeal diphtheria.

In another case the mother stated that the patient, aged five, had a serious illness at two years of age. He never seemed to learn to speak properly.

The prognosis is not bad except in the cases of young children below three. These patients are more likely to suffer from nasal obstruction and mastoid complications.

The administration of antitoxin is of little benefit by itself. The nose must be syringed with an alkaline lotion several times a day, and a spray of hydrogen peroxide and glycerine, equal parts, must be used every hour or two. In cases where the swelling of the mucous membrane is marked a solution of grs. xx. to the ounce, silver nitrate, may be brushed over the surface, or ʒss. of chloral hydrate may be added to each ounce of the spray. Pyocyanase has not proved of any value.

Swabs taken at intervals of a few days show that the organisms disappear from the discharge in less than a week. Treatment is continued until the discharge has ceased entirely.

It is interesting to note that even with a profuse nasal discharge cultures from the fauces and conjunctiva only rarely show the organism.

Every case of chronic nasal diphtheria should be notified.

